# Belladonna in Hooping Cough

**Published:** 1857-10

**Authors:** L. Turnbull

**Affiliations:** Pamphlet, Philadelphia


*Belladonna in Hooping Cough.* By Dr. L. Turnbull, (Pamphlet, Philadelphia, 1855.—* * * "In the twenty cases cured by the use of the Belladonna, the cough and hoop returned in a few cases on exposure to cold, or in disagreeable, windy weather; but, by combining the extract with syrup of ipecacuanha, a few drops soon checked the cough and hoop; in only one case out of this number was it complicated with inflammation of the lungs, and this case recovered. "The average duration of my twenty cases was ten days, after the hoop had commenced, when the case was free from complications, which shows the great advantage of this treatment. The ordinary duration of the disease, when treated in the usual manner, is from one and a half to three and a half months; even by prussic acid, or the application of Nitrate of Silver, the average is given from two to three weeks."—*Half Yearly Abstract of the Medical Sciences.*

